# Risk factors for postpartum readmission: a prediction model in Iranian pregnant women

**DOI:** 10.1186/s12884-024-06663-0

**Published:** 2024-07-06

**Authors:** Mojgan Firouzbakht, HossinAli Nikbakht, Shabnam Omidvar

**Affiliations:** 1 Department of Nursing- Midwifery, Comprehensive Health Research Center, Isalamic Azad University, Babol Branch, Iran; 2 Population, Family and Spiritual Health Research Center, Department of Biostatistics and Epidemiology, School of Public Health, Health Research Institute &, Babol University of Medical Sciences, Babol, Iran; 3 Social Determinants of Health Research Center, Health Research Institute, Babol University of Medical Sciences, Babol, Iran

**Keywords:** Readmission, Postpartum, Complications

## Abstract

**Background:**

Postpartum readmissions (PPRs) are an important indicator of maternal postpartum complications and the quality of medical services and are important for reducing medical costs. The present study aimed to investigate the risk factors affecting readmission after delivery in Imam Ali Hospital in Amol, Iran.

**Methods:**

This retrospective cohort study was conducted on the mothers who were readmitted after delivery within 30 days, at Imam Ali Hospital (2019–2023). The demographic and obstetrics characteristics were identified through the registry system. Univariate and multivariate logistic regressions with odds ratios (ORs) and 95% CIs were carried out. To identify the most important variables by machine learning methods, a random forest model was used. The data were analyzed using SPSS 22 software and R (4.1.3) at a significant level of 0.05.

**Results:**

Among 13,983 deliveries 164 (1.2%) had readmission after delivery. The most prevalent cause of readmission after delivery was infection (59.7%). The chance of readmission for women who underwent elective cesarean section and women who experienced labor pain onset by induction of labor was twice and 1.5 times greater than that among women who experienced spontaneous labor pain, respectively. Women with pregnancy complications had more than 2 times the chance of readmission. Cesarean section increased the chance of readmission by 2.69 times compared to normal vaginal delivery.

**Conclusion:**

The method of labor pain onset, mode of delivery, and complications during pregnancy were the most important factors related to readmission after childbirth.

## Background

Two-thirds of maternal deaths in developing countries occur after childbirth, and 80% of them occur in the first week after childbirth [1, 2]. According to the mortality rate of mothers in the puerperium, this period is considered to be important [3, 4]. The quality of maternal care during delivery and immediately after delivery is recognized as crucial for improving maternal care [5, 6]. Readmission after delivery is one of the mothers' morbidity indicators [7], as is maternal quality of care during delivery and postpartum [8]. Readmission refers to the unplanned or emergency return of the patient within a certain time after discharge from the hospital [9]. It is a criterion for determining the quality of health and medical care [10]. In addition, hospital readmissions have important economic consequences and may account for up to 20% of total healthcare costs [11].

Readmission within a short period after discharge from the hospital can indicate a deficiency in the care provided during admission to the hospital or in the follow-up care after discharge. For example, postsurgical infection may occur due to inadequate antibiotic prophylaxis, improper antiseptic techniques, the presence of a foreign body, or early discharge from the hospital. Moreover, there was a lack of adequate follow-up care due to social determinants (race, lack of social support, unemployment and lack of access to a proper transportation system). However, not all readmissions reflect deficiencies in care at the time of admission or deficiencies in the follow-up care system. The majority of these cases occur due to the onset of new illnesses unrelated to hospitalization or the progression of chronic disease, regardless of the type of care provided. It is estimated that only 9–48% of readmissions are associated with substandard care during hospitalization, such as a lack of complete treatment, unstable conditions at the time of discharge, or inadequate post discharge care[10, 12].

Although the hospital readmission rate is an indicator of quality [10], the postpartum readmission rate is not considered a quality indicator in maternity care [8]. Most obstetric complications occur while the mother is still in the hospital. Considering that the physiology of pregnancy continues for several months, a large number of complications related to pregnancy or childbirth may occur after discharge, requiring readmission [13].

During the last two decades, the total rate of readmission after delivery has been reported to be 1–2% [7, 14–17]. The rate was significantly greater among women who underwent cesarean section than among those who underwent NVD [9, 18, 19]. The main reasons for readmission after childbirth are bleeding, infection and high blood pressure [15, 19]. The majority of postpartum hospitalizations are readmissions of women who have given birth in THE hospitals [20]. Over the past decade, postpartum readmissions have increased by 27% (2% of all childbirths) [3], accounting for approximately 18% of all severe maternal complications [13, 21]. There are few related studies about readmission after childbirth in Iran [9, 22]. This study aimed to investigate the risk factors for readmission after childbirth in a hospital in northern Iran.

## Methods

### Design

This was a retrospective cohort study. The data of all pregnant women who gave birth from April 2019 to March 2023 at Imam Ali Hospital, Amol, Iran, were assessed.

### Data collection

All the information was accessed through the registry system of the Ministry of Health (Iman). The information of all the women referred to maternity hospitals for delivery (gestational age > 26 weeks), throughout the country was recorded from the time of admission to the maternity hospital, during delivery and until 2 h after birth The following information was collected for the present study: maternal characteristics including age, education level, place of residence (urban–rural), insurance status, BMI, number of pregnancies, method of labor pain onset (spontaneous, induction and elective cesarean section), high-risk pregnancy status (yes, no), pregnancy complications (diabetes, gestational hypertension, preeclampsia, anemia, heart disease and other disease), risk factor for childbirth status (yes–no), type of delivery risk factor (rupture of the membranes for more than 18 h, abruption placenta, stained meconium), birth intervention status (yes–no), type of intervention (episiotomy), mode of delivery (NVD, cesarean section), adverse events after deliver (transfer of the mother to the operating room after delivery, transfer to the intensive care unit or death of the mother), and newborn weight. We included in the study all Iranian women that registered in the Iman from April 2019 to March 2023.

Mothers' readmissions after childbirth were identified by using the diagnostic codes of the International Classification of Diseases, 9th and 10th revisions, and clinical modifications (ICD-9-CM and 1cd-10-CM) [23]. Postpartum hospitalization (i.e., readmission) was assessed using the fifth digit "4" in the ICD-9-CM codes for primary or secondary pregnancy-related complications. The ICD-9-CM v24 code is listed for each diagnosis, and related group codes were identified by postnatal diagnosis [13].

### Data analysis

The patients' descriptive information was reported using the mean and standard deviation or by numerical value and percentage. Binary logistic regression analysis was used to investigate separately (crud effects) and simultaneously (adjusted effects) the relationships between variables predicting mothers' readmission in the postpartum period. Since some factors can affect this relationship, these variables were considered for adjustment in the final analysis. Variables that affected the relationships between predictive variables were considered for adjustment in the final analysis. The backward method was used in the multivariate analysis, and the remaining variables in the final model are presented. The odds ratio (OR) and 95% confidence interval (CI) were used to determine the effect size in the model. Furthermore, the random forest model was also used to identify the most important variables. In forest models, one of the most important indicators is the Gini coefficient index, which is used to identify the importance of variables for predicting the readmission of mothers in the postpartum period. The data were analyzed using SPSS 22 software and R (4.1.3). The significance level for the tests was considered < 0.05.

## Results

A total of 13,983 deliveries were performed in the hospital between 2019 and 2023. In the present study, the mean and standard deviation of the ages of the pregnant women were 29.53 ± 6.02 years, ranging from 13 to 52 years. A total of 97.4% of the patients had Iranian citizenship, and 66.7% were city residents. More than half of the deliveries were by cesarean Sect. (56.3%). The onset of labor pain was induced in 48.3% of patients. Gestational diabetes and preeclampsia were observed in 5.5% and 1.4%, respectively.

Delivery risk factors and pregnancy complications were reported in 13.6% and 10.8%, respectively. Delivery intervention and delivery complications were observed in 36.4% and 0.4%, respectively.

The rate of readmission after childbirth was 1.2%. Moreover, the reasons for readmission were as follows: postpartum bleeding, 10.4%; infection, 59.7%; headache, 11.6%; episiotomy complications, 8.5%; and incision problems (i.e., hematoma and opening), 10.9%. The patient characteristics are presented in Table 1.

**Table 1:** Demographics and obstetrics characteristics of admitted women in the maternity hospital (Imam Ali), 2019-2023

Characteristics		Total (*N*=13893) N (%)	Readmission	
			No(*n*=13729) N (%)	Yes (*n*=164) N (%)
Age(year)	<35	11550 (83.1)	11416(98.8)	134(1.2)
	≥ 35	2343 (16.9)	2313(98.7)	30(1.3)
Education	diploma	9738 (70.1)	9620(98.8)	118(1.2)
	University	4155 (29.9)	4109(98.9)	46(1.1)
Insurance	No	1144 (8.2)	1133(99.0)	11(1.0)
	Yes	12749 (91.8)	12596(98.8)	153(1.2)
Residence	City	9271 (66.7)	9164(98.8)	107(1.2)
	Village	4622 (33.3)	4565(98.8)	57(1.2)
BMI^a^	<25	5700 (41.0)	5640(98.9)	60(1.1)
	≥ 25	822 (5.9)	812(98.8)	10(1.2)
Gravidity	1	4884 (35.2)	4827(98.8)	57(1.2)
	2-3	7748 (55.8)	7659(98.9)	89(1.1)
	≥ 4	1261 (9.1)	1243(98.6)	18(1.4)
Onset of Labor Pain	Spontaneous	4406 (31.7)	4371(99.2)	35(0.8)
	Induction	6710 (48.3)	6629(98.8)	81(1.2)
	Elective Cesarean section	2777 (20.0)	2729(98.3)	48(1.7)
Gestational diabetes	No	13133 (94.5)	12989(98.9)	144(1.1)
	Yes	760 (5.5)	740(97.4)	20(2.6)
Preeclampsia	No	13702 (98.6)	13545(98.9)	157(1.1)
	Yes	191 (1.4)	184(96.3)	7(3.7)
Pregnancy complications	No	12398 (89.2)	12269(99.0)	129(1.0)
	Yes	1495 (10.8)	1460(97.7)	35(2.3)
Delivery risk	No	12008 (86.4)	11866(98.8)	142(1.2)
	Yes	1885 (13.6)	1863(98.8)	22(1.2)
Delivery complication	No	13842 (99.6)	13678(98.8)	164(1.2)
	Yes	51 (0.4)	51(100.0)	0(0.0)
Delivery intervention	No	8842 (63.6)	8722(98.6)	120(1.4)
	Yes	5051 (36.4)	5007(99.1)	44(0.9)
Mode of Delivery	Normal vaginal	6074 (43.7)	6037(99.4)	37(0.6)
	Cesarean section	7819 (56.3)	7692(98.4)	127(1.6)
newborn weight	<2500	1456 (10.5)	1433 (98.4)	23(1.6)
	2500-4000	11865 (85.4)	11729(98.9)	136(1.1)
	>4000	572 (4.1)	567(99.1)	5(0.9)

^a^Other cases: Missing

Univariate and multivariate regression analyses of the variables influencing readmission after childbirth revealed that the labor pain onset method and pregnancy complications were related to readmission. Therefore, these two variables were identified as independent and strong predictors for readmission. (Table 2).

**Table 2:** Univariate logistic regression analysis in readmission after childbirth

Characteristics	Sub groups	B(SE)	OR	%95CI	*P*-value
Age(year)	<35	R 0.10(0.20)	1.10	0.74 to 1.64	0.623
	≥ 35				
Education	diploma and lower	R -0.09(0.17)	0.91	0.64 to 1.28	0.601
	University				
Insurance	No	R 0.22(0.31)	1.25	0.67 to 2.31	0.475
	Yes				
Residence	City	R 0.372 (0.722)	1.06	0.77 to 1.47	0.684
	Village				
BMI	<25	R 0.14 (0.34)	1.15	0.59 to 2.27	0.670
	≥ 25				
Gravidity	1	R -0.01(0.17)0.20(0.27)	0.98 1.22	0.70 to 1.37 0.71 to 2.09	0.625 0.454
	2-3				
	≥ 4				
Onset of Labor Pain	Spontaneous	R 0.42(0.20) 0.78(0.22)	1.52 2.19	1.02 to 2.27 1.41 to 3.40	0.038* <0.001**
	Induction				
	Elective Cesarean section				
£Pregnancy complications	No	R 0.82 (0.19)	2.28	1.56 to 3.32	<0.001**
	Yes				
£Gestational diabetes	No	R 0.89 (0.24)	2.43	1.51 to 3.91	<0.001**
	Yes				
£Preeclampsia	No	R 1.18(0.39)	3.28	1.51 to 7.09	0.003*
	Yes				
Delivery risk	No	R -0.01(0.23)	0.98	0.62 to 1.55	0.954
	Yes				
Delivery intervention	No	R -0.44 (0.17)	0.63	0.45 to 0.90	0.011*
	Yes				
Mode of Delivery	Normal vaginal	R 0.99 (0.18)	2.69	1.86 to 3.89	<0.001**
	Cesarean section				
Newborn weight	<2500	R -0.32(0.22) -0.59(0.49)	0.72 0.54	0.46 to 1.12 0.20 to 1.45	0.152 0.227
	2500-4000				
	>4000				

£: These variables just entered in the univariate regression

*P<0.05

**P<0.001

Univariate and multivariate regression analyses revealed that women with pregnancy complications had a 2.28-fold greater chance of readmission (95% CI: 1.56 to 3.32, *P* < 0.001) and a 2.05-fold greater chance of readmission (95% CI: 1.15 to 3.65, *P* < 0.014), respectively. The most pregnancy complications were diabetes (5.5%), and preeclampsia (1.4%). Univariate regression analysis Women with gestational diabetes and preeclampsia had 2.43-fold (95% CI: 1.51 to 3.91, *P* < 0.001) and 3.28-fold (95% CI: 1.51 to 7.09, *P* = 0.003) greater chances of readmission, respectively.

There were more than 2 folds (95% CI: 1.41 to 3.40, *P* < 0.001) among women who had elective cesarean sections and 1.5 folds (95% CI: 1.02 to 2.27, *P* = 0.038) among women who had induction of labor greater chances of readmission compared to women with spontaneous labor pain. (Table 3).

**Table 3:** Multivariate logistic regression analysis (adjusted effects) in readmission after childbirth (backward model)

Characteristics	Sub groups	B(SE)	OR	%95CI	*P*-value
Onset of Labor Pain	Spontaneous	R 0.68(0.29) 0.33(0.48)	1.99 1.39	1.11 to 3.54 0.53 to 3.61	0.020* 0.493
	Induction				
	Elective Cesarean section				
Pregnancy complication	No	R 0.72(0.29)	2.05	1.15 to 3.65	0.014*
	Yes				

Variables included in the Backward model: Age, Education, Insurance, Residence, BMI, Gravidity, onset of Labor Pain, Complication pregnancy, Delivery risk, Delivery intervention, Delivery type, and Baby weigh

*P<0.05

Moreover, the results showed that compared with NVD, cesarean delivery significantly increased the chance of readmission by 2.69 times (95% CI: 1.86 to 3.89, *P* = 0.003). Women who underwent obstetric interventions such as episiotomy had a 37% lower chance of readmission.

In the present study, mothers older than 35 years and with a BMI greater than 25 had a greater chance of readmission (95% CI: 0.76 to 1.64, *P* = 0.623, OR = 1.10 and 95% CI: 0.59 to 2.27, *P* = 0.67, OR = 1.15, respectively). However, this relationship was not significant.

**Fig. 1:**
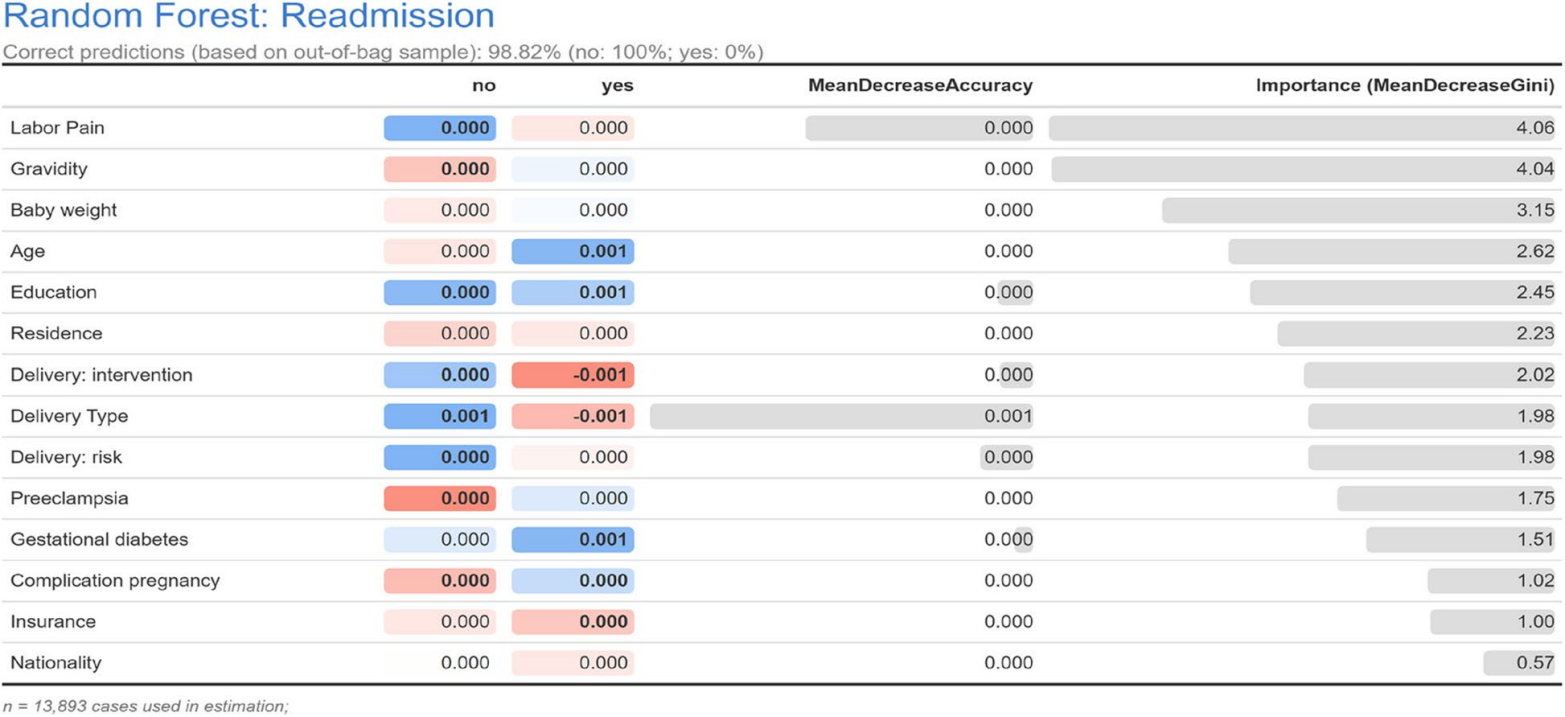
Random forest model to predict the most important factors in the readmission of mothers in the postpartum period

The results of the random forest model based on the variables in the study showed that all the variables were important, and none of the variables were removed from the model. Figure 1 shows the importance estimated by the conditional forest algorithm. The importance of variables based on the Gini importance index (mean decrease Gini) showed that the most important variable was the method of labor pain onset (4.06%), and gravidity (4.04%) and newborn weight (3.15%) were identified in the next categories for predicting readmission in the postpartum period. (Fig. 1).

### Discussion

This study aimed to determine the factors related to readmission after childbirth. The method of labor pain onset, mode of delivery, and pregnancy complications were identified as the most important factors related to readmission to the hospital. In the present study, women who underwent cesarean section and induced labor were more likely to be readmitted after delivery than women who underwent spontaneous delivery. Studies have reported that increasing the length of labor [24], prolonging the rupture of membranes, increasing the chance of postpartum hemorrhages [25], and increasing perineal rupture due to induction may increase the rate of readmission [26]. In other studies, the mode of delivery [27–30], induction of labor [24], and pregnancy complications [16, 31–33] have been reported to be factors related to readmission.

In the present study, performing an episiotomy was associated with a decrease in the rate of readmission after childbirth. Performing an episiotomy can reduce severe perineal tears and therefore affect the rate of readmission. Another study reported that performing an episiotomy was associated with a reduction in perineal tearing [26].

The rate of readmission after childbirth reported in other studies ranged from 1.2 to 16.2% [17, 34–36]. This rate in our study was 1.2%. However, referring women to other hospitals or other cities after giving birth can also affect the reported rate.

The results of the study revealed a greater chance of readmission in mothers with a history of diabetes and hypertension during pregnancy. Several studies have shown that these complications are risk factors for readmission after childbirth [31, 33, 32, 37]. Women with diabetes, especially diabetes type 1, as well as people with poor glycemic control during pregnancy, had a greater chance of being readmitted, especially due to infection. These results show the importance of multidisciplinary care for mothers and the control of complications during pregnancy [2].

Another related factor to readmission was the mother's age over 35 years. Researcher reported, women over 35 years were more likely to be re-hospitalized after childbirth due to severe maternal complications. Older age was associated with higher complications during pregnancy, readmission after delivery, and adverse neonatal outcomes [38].

The results of the study indicated that the chance of readmission among obese women was greater than that among women with a normal BMI. However, the difference was not statistically significant. The results of other studies [8, 30] showed that women with a high BMI had a greater risk for diabetes and readmission due to infection.

In the present study, the main causes of readmission were surgical incision problems (i.e., infection, wound dehiscence and hematoma) and postpartum bleeding. These findings are in line with numerous studies concluding that bleeding, infection, and hypertension are the main reasons for readmission after childbirth [15, 16, 19].

A mother's readmission within a short interval after delivery is stressful for the mother, newborn, and family. Identifying the factors related to re-hospitalization can be considered in care protocols for mothers after childbirth to minimize severe complications after childbirth and reduce maternal mortality. The World Health Organization has recommended postpartum care at 24 h, 3 days (24 to 72 h after delivery), 7 to 14 days, and six weeks after delivery [19, 39]. Although all readmissions are not preventable, identifying women at risk for more preventive care is essential. Recent clinical recommendations emphasize improving postpartum care (as the fourth trimester of pregnancy).

According to the recommendation of the ACOG, "to optimize the maternal and child health, postpartum care should be considered as an ongoing process and health services and supports must be based on individual needs” [40]. A standard visit may be appropriate for low-risk women, but high-risk women require special care [41].

According to the definition of quality of patient discharge from the hospital (as the experience of patients with sufficient education and resources at the time of discharge) and its association with a lower readmission rate among the general population [42, 43], providing suitable protocols for patient discharge and comprehensive assessments of the individual and educational interventions are recommended [15].

### Strengths and limitations

There were study limitations in the interpretation of the results. First, although information related to childbirth was collected through a comprehensive information system, information records were missing, which can affect the results. Second, due to the closeness of the city (Amol) to the capital city (Tehran) and other cities that have more advanced academic centers, it is possible that several cases of readmission were readmitted to other hospitals in nearby cities.

One of the strengths of this study was the large sample size. On the other hand, all the data of the study were related to one hospital, which can reduce the dispersion in the method of data recording. To the best of our knowledge, the present study is the first study with a large sample that aimed to determine the factors related to readmission in Iran.

## Conclusion

Our results highlighted that the most important factors related to readmission after childbirth were the method of labor pain onset, mode of delivery and complications during pregnancy. All the risk factors we identified were mostly related to demographic and obstetric characteristics. Improving the quality of care during pregnancy as well as postpartum care for high-risk pregnant women seems to be the golden key to reducing readmission. Additionally, interventions such as women's education regarding early warning signs after childbirth to prevent potentially life-threatening complications are necessary.

### Clinical application

Since a considerable portion of maternal morbidity and mortality are related to the postpartum period, understanding the importance of the quality of inpatient and outpatient postpartum care in improving maternal outcomes is essential. Identifying at-risk women and designing purposeful interventions might reduce mortality and morbidity.

## Data Availability

Data is supplementary information files.

## Abbreviations

PPRs: Postpartum readmissions
ORs: Odds ratios
CI: Confidence interval
ICD-9-CM and 1cd-10-CM: International Classification of Diseases, 9th and 10th revisions
NVD: Normal vaginal delivery

## References

[1] World Health Organization. Making pregnancy safer: the critical role of the skilled attendant: a joint statement by WHO, ICM and FIGO. World health organization; 2004.

[2] Nieburg P (2012). Improving Maternal Mortality and other aspects of women’s health.

[3] Pinheiro RL, Areia AL, Mota Pinto A, Donato H (2019). Advanced maternal age: adverse outcomes of pregnancy, a meta-analysis. Acta Med Port.

[4] Kassebaum NJ, Barber RM, Bhutta ZA, Dandona L, Gething PW, Hay SI (2016). Global, regional, and national levels of maternal mortality, 1990–2015: a systematic analysis for the global burden of disease study 2015. The Lancet.

[5] McKee KS, Akobirshoev I, McKee M, Li FS, Mitra M (2023). Postpartum hospital readmissions among massachusetts women who are deaf or hard of hearing. J Womens Health (Larchmt).

[6] Petersen EE, Davis NL, Goodman D, Cox S, Mayes N, Johnston E (2019). Vital signs: pregnancy-related deaths, United States, 2011–2015, and strategies for prevention, 13 states, 2013–2017. Morb Mortal Wkly Rep.

[7] Nam JY, Park E-C (2020). The relationship between severe maternal morbidity and a risk of postpartum readmission among Korean women: a nationwide population-based cohort study. BMC Pregnancy Childbirth.

[8] Clapp MA, Robinson JN, Little SE (2017). The relationship between the rising cesarean delivery and postpartum readmission rates. J Perinatol.

[9] Eslamimoghadam F, Aliabadi F, Afrashteh S, Abbasi M, Ahmadli R, Mohammadbeigi A. Prevalence of post-cesarean readmission and its related factors in women delivered by cesarean of Qom hospitals, 2017,(Iran).

[10] Combs CA, Goffman D, Pettker CM, SfM-F Medicine, Committee Q (2022). Society for maternal-fetal medicine special statement: a critique of postpartum readmission rate as a quality metric. Am J Obstet Gynecol.

[11] Jencks SF, Williams MV, Coleman EA (2009). Rehospitalizations among patients in the medicare fee-for-service program. N Engl J Med.

[12] Benbassat J, Taragin M (2000). Hospital readmissions as a measure of quality of health care: advantages and limitations. Arch Intern Med.

[13] Callaghan WM, Creanga AA, Kuklina EV (2012). Severe maternal morbidity among delivery and postpartum hospitalizations in the United States. Obstet Gynecol.

[14] Aziz A, Gyamfi-Bannerman C, Siddiq Z, Wright JD, Goffman D, Sheen JJ (2019). Maternal outcomes by race during postpartum readmissions. American journal of obstetrics and gynecology..

[15] Belfort MA, Clark SL, Saade GR, Kleja K, Dildy GA, Van Veen TR (2010). Hospital readmission after delivery: evidence for an increased incidence of nonurogenital infection in the immediate postpartum period. Am J Obstet Gynecol.

[16] Clapp MA, Little SE, Zheng J, Robinson JN (2016). A multi-state analysis of postpartum readmissions in the United States. Am J Obstet Gynecol.

[17] Girsen AI, Sie L, Carmichael SL, Lee HC, Foeller ME, Druzin ML (2020). Rate and causes of severe maternal morbidity at readmission: California births in 2008–2012. J Perinatol.

[18] Girsen AI, Leonard SA, Butwick AJ, Joudi N, Carmichael SL, Gibbs RS (2022). Early postpartum readmissions: identifying risk factors at birth hospitalization. AJOG Global Reports.

[19] Symonds NE, Vidler M, Wiens MO, Omar S, English LL, Ukah UV (2023). Risk factors for postpartum maternal mortality and hospital readmission in low-and middle-income countries: a systematic review. BMC Pregnancy Childbirth.

[20] MacDorman MF, Declercq E (2016). Trends and characteristics of United States out-of-hospital births 2004–2014: new information on risk status and access to care. Birth.

[21] Johnson PD, Duzyj CM, Howell EA, Janevic T (2019). Patient and hospital characteristics associated with severe maternal morbidity among postpartum readmissions. J Perinatol.

[22] Moudi F, Moudi Z, Yaghmaei M (2007). Relationship between type of delivery and cause of hospitalization in postpartum period. Journal title.

[23] Kuklina EV, Whiteman MK, Hillis SD, Jamieson DJ, Meikle SF, Posner SF (2008). An enhanced method for identifying obstetric deliveries: implications for estimating maternal morbidity. Matern Child Health J.

[24] Grobman WA, Sandoval G, Reddy UM, Tita ATN, Silver RM, Mallett G (2020). Health resource utilization of labor induction versus expectant management. Am J Obstet Gynecol.

[25] Abecassis A, Wainstock T, Sheiner E, Miodownik S, Pariente G. Risk factors for early postpartum hemorrhage: A retrospective, population‐based, cohort analysis.Int J Gynecol Obstet. 2024;00:1–7. 10.1002/ijgo.15414. 10.1002/ijgo.15414 38321817

[26] Seijmonsbergen-Schermers AE, Peerdeman KM, van den Akker T, Titulaer LM, Roovers J-P, Peters LL, et al. Differences in rates of severe perineal trauma between midwife-led and obstetrician-led care in the Netherlands: A nationwide cohort study. Heliyon. 2024;10(2). 10.1016/j.heliyon.2024.e24609 PMC10835235 38312656

[27] Harrison MS, Pasha O, Saleem S, Ali S, Chomba E, Carlo WA (2017). A prospective study of maternal, fetal and neonatal outcomes in the setting of cesarean section in low-and middle-income countries. Acta Obstet Gynecol Scand.

[28] Oladapo OT, Lamina MA, Sule-odu AO (2007). Maternal morbidity and mortality associated with elective caesarean delivery at a university hospital in Nigeria. Australian New Zealand J Obstet Gynaecol.

[29] Ade-Conde JA, Alabi O, Higgins S, Visvalingam G (2011). Maternal post natal hospital readmission-trends and association with mode of delivery. Ir Med J.

[30] Jauk V, Saade G, Boggess K, Longo S, Clark E, Esplin S (2019). Incidence and risk factors for hospital readmission or unexpected visits in women undergoing unscheduled cesarean delivery. Am J Perinatol.

[31] Stamilio DM, Beckham AJ, Boggess KA, Jelovsek JE, Venkatesh KK (2021). Risk factors for postpartum readmission for preeclampsia or hypertension before delivery discharge among low-risk women: a case-control study. Am J Obstet Gynecol MFM.

[32] Mourad M, Wen T, Friedman AM, Lonier JY, D'Alton ME, Zork N (2020). Postpartum readmissions among women with diabetes. Obstet Gynecol.

[33] Wen T, Yu VX, Wright JD, Goffman D, Attenello F, Mack WJ (2020). Postpartum length of stay and risk for readmission among women with preeclampsia. J Matern Fetal Neonatal Med.

[34] Liu S, Heaman M, Joseph KS, Liston RM, Huang L, Sauve R (2005). Risk of maternal postpartum readmission associated with mode of delivery. Obstet Gynecol.

[35] Sharvit M, Rubinstein T, Ravid D, Shechter-Maor G, Fishman A, Biron-Shental T (2014). Patients with high-risk pregnancies and complicated deliveries have an increased risk of maternal postpartum readmissions. Arch Gynecol Obstet.

[36] Igbaruma S, Olagbuji B, Aderoba A, Kubeyinje W, Ande B, Imarengiaye C (2016). Severe maternal morbidity in a general intensive care unit in Nigeria: clinical profiles and outcomes. Int J Obstet Anesth.

[37] Bruce KH, Anderson M, Stark JD (2021). Factors associated with postpartum readmission for hypertensive disorders of pregnancy. Am J Obstet Gynecol MFM.

[38] Wen T, Overton EE, Sheen JJ, Attenello FJ, Mack WJ, D'Alton ME (2021). Risk for postpartum readmissions and associated complications based on maternal age. J Matern Fetal Neonatal Med.

[39] Word Health Organization. Recommendations on Postnatal Care of the Mother and Newborn. Available from: https://www.ncbi.nlm.nih.gov/books/NBK190090/. Geneva2013 Oct. Executive summary. 24624481

[40] Care OP (2018). ACOG committee opinion No. 736. American college of obstetricians and gynecologists. Obstet Gynecol.

[41] Horwitz MM, Molina RL, Snowden JM (2019). Postpartum care in the United States: new policies for a new paradigm. Obstet Anesth Dig.

[42] Henke RM, Karaca Z, Jackson P, Marder WD, Wong HS (2017). Discharge planning and hospital readmissions. Med Care Res Rev.

[43] Oh EG, Lee HJ, Yang YL, Kim YM (2021). Effectiveness of discharge education with the teach-back method on 30-day readmission: a systematic review. J Patient Saf.

